# CMOS-Compatible Fabrication for Photonic Crystal-Based Nanofluidic Structure

**DOI:** 10.1186/s11671-017-1849-7

**Published:** 2017-02-09

**Authors:** Wang Peng, Youping Chen, Wu Ai, Dailin Zhang, Han Song, Hui Xiong, Pengcheng Huang

**Affiliations:** 1 School of Mechanical Science and Engineering, Huazhong University of Science and Technology, Wuhan, 430074 China; 2 Department of Electrical and Computer Engineering, University of Illinois at Urbana-Champaign, Urbana, Illinois 61801 USA; 3 Micro and Nanotechnology Laboratory, University of Illinois at Urbana-Champaign, Urbana, Illinois 61801 USA; 4 School of Mechanical and Electronic Engineering, Wuhan University of Technology, Wuhan, 430070 China; 5 Flexible Electronics Research Center, Huazhong University of Science and Technology, Wuhan, 430074 China

**Keywords:** Photonic crystal, PC-based nanofluidic structure, CMOS-compatible process, Periodical Si_3_N_4_ gratings

## Abstract

Photonic crystal (PC)-based devices have been widely used since 1990s, while PC has just stepped into the research area of nanofluidic. In this paper, photonic crystal had been used as a complementary metal oxide semiconductors (CMOS) compatible part to create a nanofluidic structure. A nanofluidic structure prototype had been fabricated with CMOS-compatible techniques. The nanofluidic channels were sealed by direct bonding polydimethylsiloxane (PDMS) and the periodic gratings on photonic crystal structure. The PC was fabricated on a 4-in. Si wafer with Si_3_N_4_ as the guided mode layer and SiO_2_ film as substrate layer. The higher order mode resonance wavelength of PC-based nanofluidic structure had been selected, which can confine the enhanced electrical field located inside the nanochannel area. A design flow chart was used to guide the fabrication process. By optimizing the fabrication device parameters, the periodic grating of PC-based nanofluidic structure had a high-fidelity profile with fill factor at 0.5. The enhanced electric field was optimized and located within the channel area, and it can be used for PC-based nanofluidic applications with high performance.

## Background

Photonic crystal (PC) is defined as a relatively high refractive index layer of modulated periodic structure, and it is surrounded by two relatively low refractive index materials. When a collimated and polarized white light projects on the surface of the periodic structure, the PC can interact strongly with the specific optical wavelength, and the electric field of PC will be redistributed. Then, the specific wavelength is reflected back with a particular angle according to the light’s incident direction, while the remained wavelengths light transmit through the PC structure. The reflected optical wavelength is regarded as optical resonance wavelength, and the electric field of PC gratings can be enhanced many times than the initial incident light. Since Yablonovitch proposed the concept of photonic crystal [1], PC have been used extensively in various fields, including PC-based waveguide [2], fiber [3], back reflector [4], and light-emitting diode [5].

With the rapid development of life science, the methods for gene detection, protein-binding analysis, small molecules, and DNA detection are urgently needed. PC-based biosensors are studied and fabricated widely. The principle for PC-based biosensors is the interaction between the liquid sample and the enhanced electric field of PC. Basically, there are two kinds of PC-based detection methods: label-free detection [6, 7] and fluorescence-enhanced detection [8–10]. However, all the PC-related devices rely on the enhanced electrical field magnitude on the surface of the PC gratings, which is known as evanescent field. The functional range of the evanescent field is under 300 nm from the PC surface [11], which means that the tremendous space of the PC-based bulk device cannot make a contribution to the detection result. Moreover, for PC-based chemical, biological, and medical applications, the analytes are either required to flow through optical sensor or bind to the surface of PC gratings by diffusion [12, 13] so as to use the evanescent field on the PC surface. With traditional bulk sensors, it may take several hours for the analytes to reach the surface of optical flow sensor due to the laminar flow [14, 15], especially in low concentration detection [16, 17]. Thus, the PC-based bulk sensors that require large volume of analytes and need a long diffusion time may not be adaptable for future detection among proteins, genes, and small molecules. However, PC-based nanofluidic sensors paved a new way for efficient and high-performance detection [18–20]. The nanofluidic channel can guarantee the detection area be confined around the evanescent region of PC structure, which can improve the sensor’s sensitivity and reduce sample volume. Also, due to the large surface to volume ratio, the analytes in the nanochannels can flow to the channel walls easily.

In this paper, a novel PC-based nanofluidic structure has been designed and fabricated with CMOS-compatible process [21]. PC-based nanofluidic structure is an important CMOS-compatible part, and it can be used to realize the lab on a Chip concept for parallel and high through-put applications. The PC-based nanofluidic structure was fabricated on a 4-in. Si wafer, with Si_3_N_4_ as the high refractive index layer and SiO_2_ as relatively low refractive index substrate. A PDMS stamp was used to directly seal the PC gratings and create the nanochannels. The PC gratings were patterned by PMMA with e-beam lithography, and then the grating patterns were transferred to a thin chrome layer. The chrome was used as hard mask to transfer the grating pattern precisely to the Si_3_N_4_ film. All the related parameters had been analyzed and calculated during the fabrication processes.

## Methods

### Principle of PC-Based Nanofluidic Structure

**Fig. 1:**
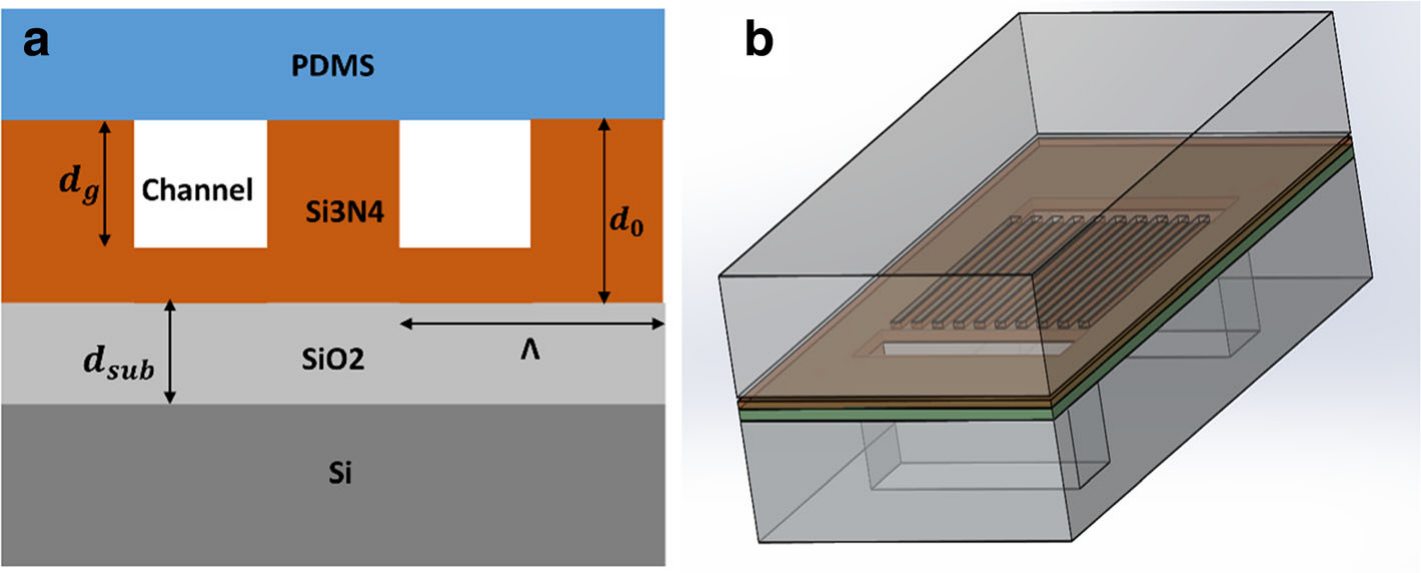
Principle of PC-based nanofluidic structure. **a** PC-based nanofluidic structure with related parameters: SiO_2_ thickness d_sub_, grating period Λ, channel depth d_g_, Si_3_N_4_ thickness d_0_. **b** Schematic diagram of PC-based nanofluidic structure

The proposed nanofluidic sensor structure is shown in Fig. 1a: the PC structure is configured by periodical Si_3_N_4_ layer as guided layer and a flat SiO_2_ layer on top of Si wafer as substrate, and PDMS is used as cladding layer to seal the PC gratings. As shown in Fig. 1b, the inlet and outlet of the nanofluidic structure were drilled from the back side of Si wafer. With this structure, the resonance wavelength energy can be largely coupled into the guided layer and reduce the leaked energy around resonance wavelength of the PC. The Si_3_N_4_ layer thickness is d_0_, and the grating depth is d_g_. The thickness of SiO_2_ layer is d_sub_, and it was used as relatively low refractive index for PC. As discussed in [22], there were several parameters influence the resonance wavelength of PC-based nanofluidic structure. The subwavelength pitch and the remained thickness of Si_3_N_4_ determine the peak wavelength position of the PC, while the grating depth d_g_ can be used to tune the PC bandwidth. The resonance wavelength generated when the collimated and polarized incident light source matches with phase-matching condition [23], as shown in Eq. (1):

1 $$ \frac{2\pi}{\lambda}{n}_{\mathrm{eff}}=\frac{2\pi}{\lambda}{n}_{\mathrm{c}} sin\theta \pm m\left(\frac{2\pi}{\varLambda}\right) $$

where λ is the incident light which couples into the PC, ${n}_{\mathrm{eff}}$ is the effective refractive index of photonic crystal, ${n}_{\mathrm{c}}$ is the refractive index of the cover layer, *θ* is the incident angle, and $m=\pm 0,1,2\dots$ is the diffractive order. In order to simplify the structure for fabrication and experiments, the incident angle is always vertical to the surface of the PC, and the fill factor of the grating is 0.5. So, the equation can be simplified as Eq. (2):

2 $$ \uplambda =\varLambda\ {n}_{\mathrm{eff}} $$

,which shows a concise relationship among resonance wavelength, PC grating pitch, and effective refractive index. The effective refractive index can be roughly regraded as weighting average refractive index of Si_3_N_4_, channel liquid, and PDMS.

### Simulation Result and Analysis

As the shape of gratings were rectangular, the gap between PC gratings can be used to fabricate channels when the gratings’ top is sealed with PDMS. With this novelty design, the PC gratings were used as nanofluidic channel. FDTD Solutions from Lumerical Inc. had been used for electromagnetic field simulation. Based on our former design method that had been published in [24], the parameters of PC-based nanofluidic structure had been optimized [25]. The gratings period was set as 400 nm, which is a reasonable value for PC fabrication with resonance wavelength located at visible wavelength range. The reason for fixing PC’s grating period is that it is difficult to tune the pitch among fabrication processes, while it is relatively easier to vary the Si_3_N_4_ thickness d_0_ and grating depth d_g_. By tuning the Si_3_N_4_ thickness and grating depth, the PC resonance wavelength can be changed according to the requirements. Then, the remained parameters that need to be optimized in FDTD is the Si_3_N_4_ thickness d_0_ and grating depth d_g_. Particle swarm optimization algorithm in FDTD had been used to select the appropriate PC structure parameters.

**Fig. 2:**
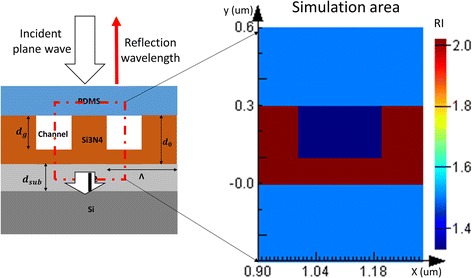
Illumination of photonic crystal-based nanofluidic structure simulation

During FDTD simulation, the incident light was a TE-polarized plane wave that ranges from 400 to 700 nm, and the direction of the incident light was perpendicular to the top surface of PC plane, as shown in Fig. 2. The fixed parameters of PC structure were grating period Λ = 400 nm, the thickness of SiO_2_ substrate d_SiO2_ = 2 μm, and the refractive indexes of Si_3_N_4_, PDMS, and channel liquid were set as 2.02, 1.45, and 1.33, respectively. Then, the thickness of Si_3_N_4_ d_0_ was varying as 500, 450, 400, 350, and 300 nm, while the grating depth d_g_ was changing as d_0_, d_0_ − 50 nm, d_0_ − 100 nm, …, 100 nm, 50 nm. By using different Si_3_N_4_ thickness d_0_ and grating depth d_g_, a series of PC structure were obtained. The electric field distribution and enhancement factor of all these PC structures were obtained by running FDTD simulation software, and the PC structure which can yield an appropriate electric field distribution and enhancement factor was chosen as our sensor structure. The related PC based FDTD simulation process can be found in our former published papers [24, 26].

The final optimized results were shown as follows: PC period Λ was 400 nm, Si_3_N_4_ thickness d_0_ was 450 nm, and grating depth d_g_ was 350 nm. With the grating depth located at 350 nm, most of the channel area was inside the active region of evanescent field, and the performance of PC based nanofluidic structure can be improved with this structure.

**Fig. 3:**
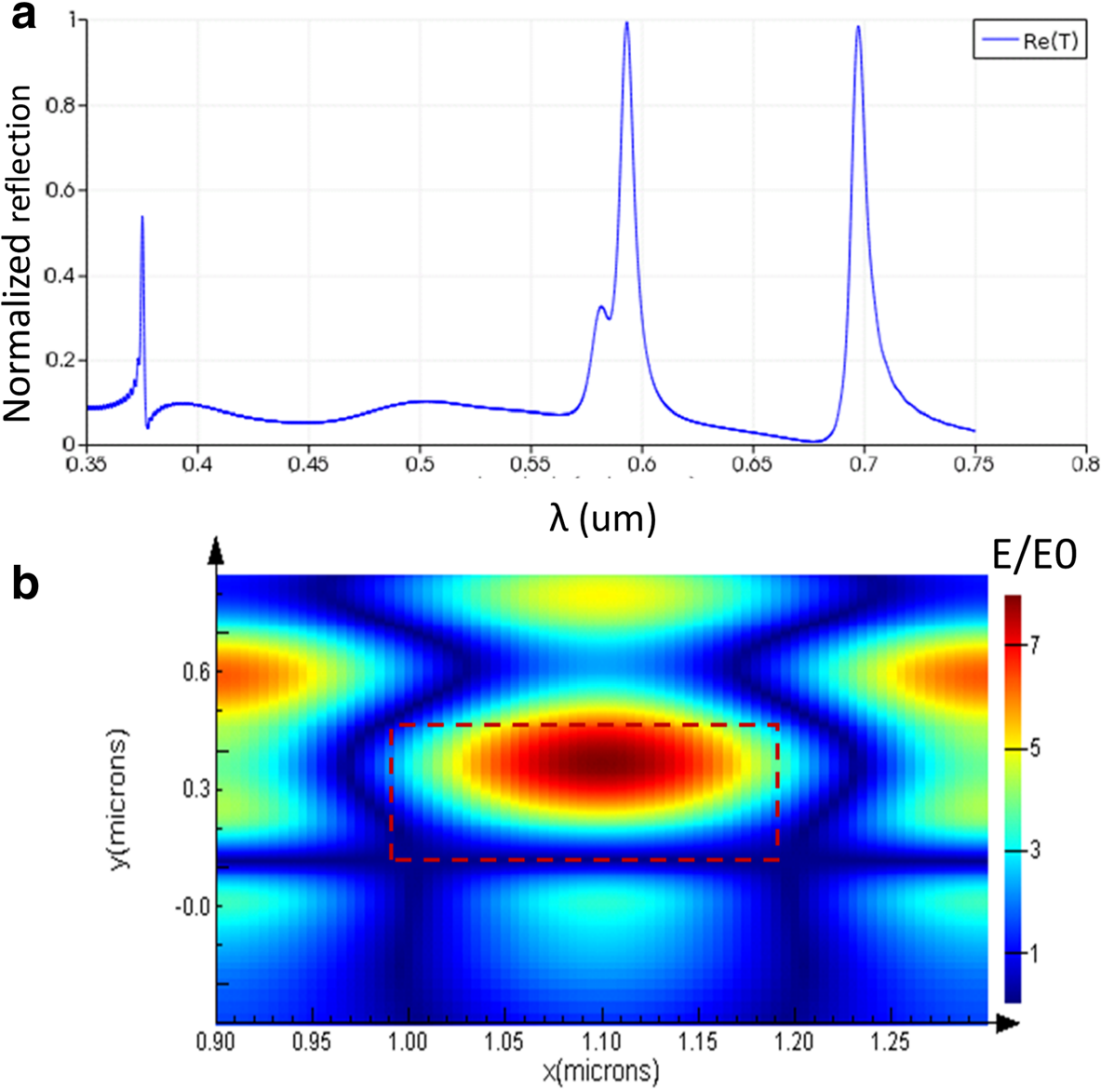
Simulation result of PC-based nanofluidic structure, TE polarized, SiO_2_ thickness d_sub_ 2 μm, grating period Λ 400 nm, channel depth d_g_ 350 nm, Si_3_N_4_ thickness d_0_ 450 nm. **a** Normalized reflection spectrum of PC, peak wavelength value (PWV) at TE00 698 nm, peak wavelength value at TE01 595 nm. **b** Electrical field enhancement of PC-based nanofluidic structure with PWV at 598 nm

As shown in Fig. 3a, when the light source is collimated and TE polarized, the PC’s spectrum has two peaks around visible range at normal incidence. The peak at 698 nm was the zero order mode TE00, and the peak at 595 nm is the first order mode TE01. In application, visible light is much easier to capture during the label-free detection. So, the first order peak was selected for label-free detection. As shown in Fig. 3b, the electric field intensity of peak at 595 nm has been obtained, and the red-dashed box represents the area in which it is used as nanochannel. It indicated that the enhanced E-field was almost confined in the channel area, which means that the electric field can interact with the sample in the channel sufficiently. The maximum electric field enhancement is more than 7 times larger than the initial incident light electric field. The former reported PC structures may have a much high maximum E-field-enhanced intensity. However, the enhanced area is usually located around the corners of the gratings or the inside part of the high refractive index layer [27, 28]. In the former structure, the enhanced E-field cannot interact with the sample thoroughly, which may degrade the performance of PC-based sensors.

## Results and Discussion

### Design Flow of PC-Based Nanofluidic Structure

**Fig. 4:**
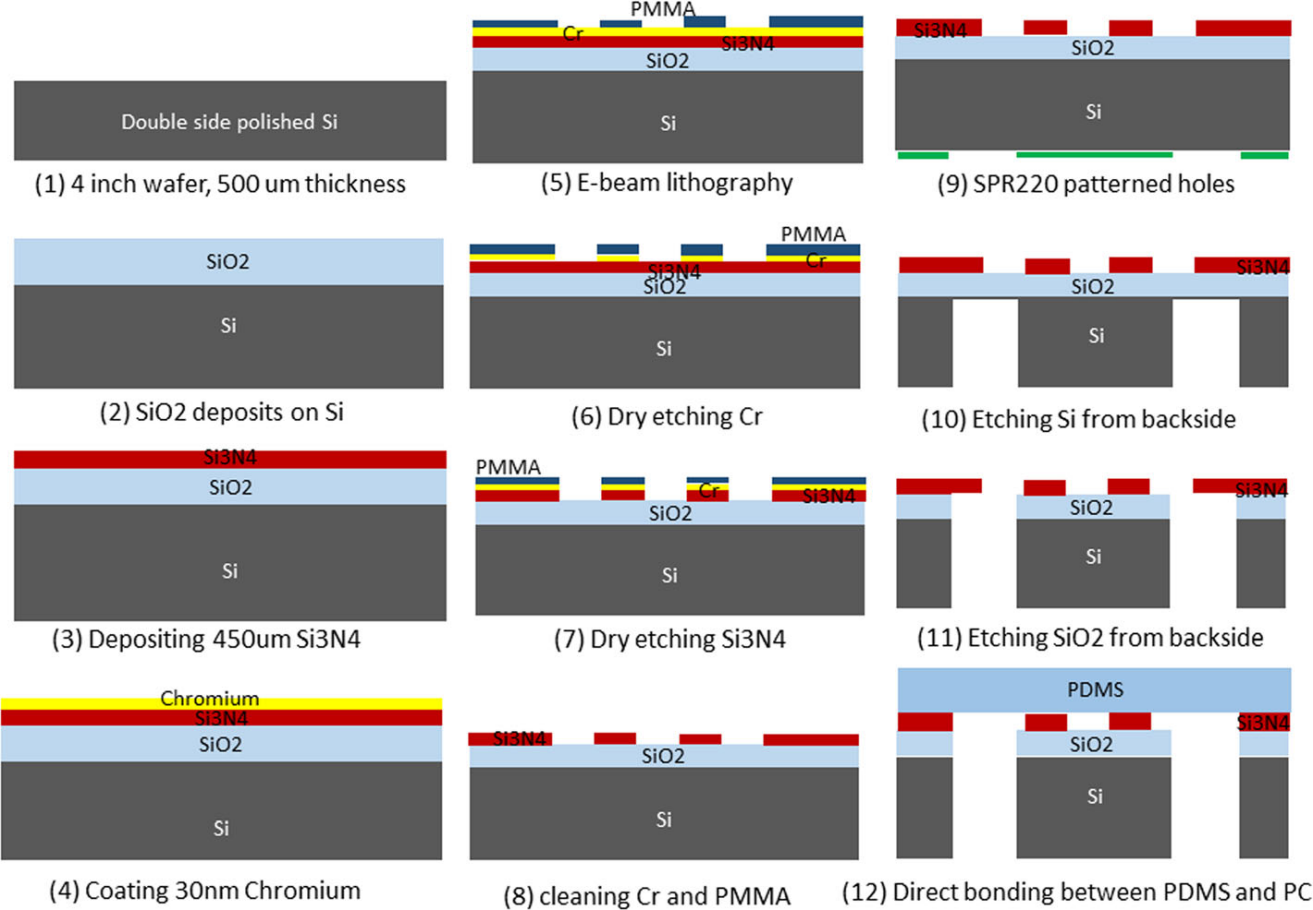
Fabrication flow process of PC-based nanofluidic structure

All the experiments were accomplished on a 4-in. Si wafer, and CMOS-compatible process has been used for fabrication. As shown in Fig. 4, the designed flow of the nanofluidic sensor is as follows: (1) Si wafer clean, (2) 2 μm SiO_2_ deposited on Si wafer as PC substrate, (3) 450 nm Si_3_N_4_ deposited on SiO_2_ layer as PC high refractive index layer, (4) 30 nm Cr coated as hard mask, (5) 2600 nm PMMA spin coated on Cr as photoresist for e-beam grating writing, (6) etching Cr layer with patterned PMMA as mask, (7) etching Si_3_N_4_ layer with etched Cr layer as mask, (8) removing PMMA and Cr, (9) patterned inlets/outlets from backside of the wafer with optical lithography, (10) etching through Si layer with SPR 220 as photoresist, (11) etching SiO_2_ with Si as mask layer, (12) directed bonding between PDMS and patterned Si wafer.

### SiO_2_ and Si_3_N_4_ Deposition Experiments

**Fig. 5:**
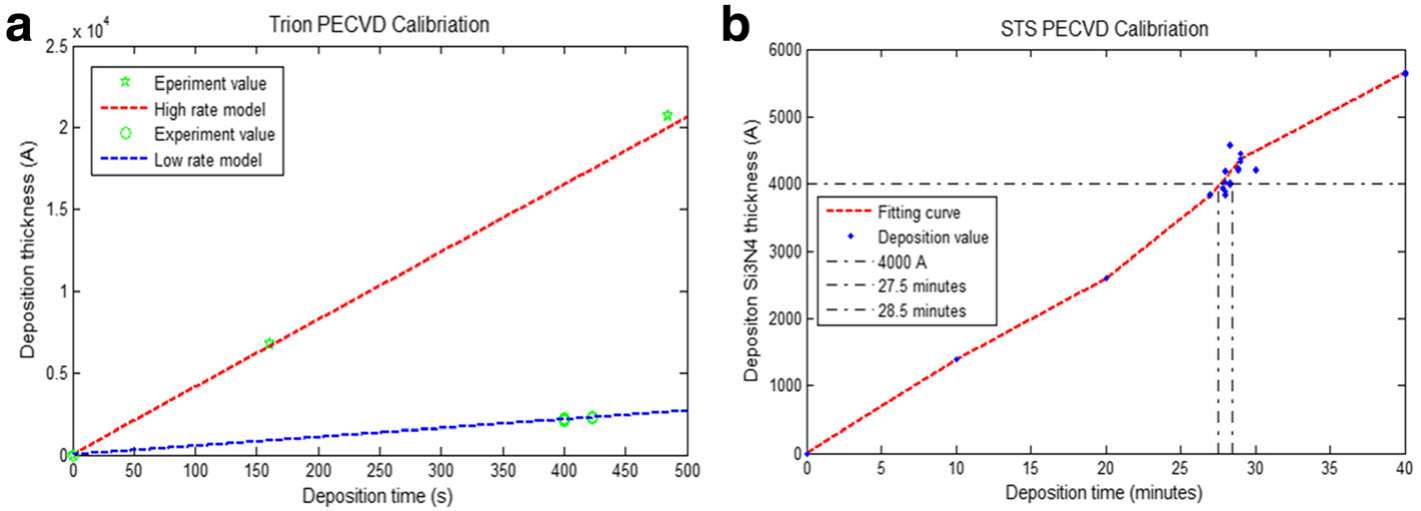
Deposition rate calibration. **a** Calibration of Trion PECVD deposition rate for SiO_2_. **b** Calibration of STS PECVD deposition rate for Si_3_N_4_

The double side polished Si wafer (100) was boron doped from Wafer World, Inc. A piranha process with 1:1 volumetric mixture of concentrated sulfuric acid (H_2_SO_4_) and hydrogen peroxide (wt. 30%) was used to clean the Si wafer at 60 °C for 10 min. Then, the wafer was rinsed by DI water and dried with N_2_ gas. For the deposition of SiO_2_, Trion PECVD (Plasma Enhanced Chemical Vapor Deposition) with high/low deposition rates were used. As shown in Fig. 5a, the high deposition rate is much faster while the film density of low deposition rate method is much higher and with less pin holes inside the SiO_2_ thin film. Since the SiO_2_ layer is 2 μm, the low deposition rate (320 Å/min, ±25 Å) was selected, with temperature at 350 °C, ICP power 102 W and gases of N_2_O, N_2_, and SiH_4_.

After the deposition of SiO_2_ film on Si wafer, a thickness of 450-nm Si_3_N_4_ film was deposited on the top of SiO_2_ film as guided mode layer. Since Si_3_N_4_ was used as the guided layer for photonic crystal, the thickness of Si_3_N_4_ has a critical influence to the resonance wavelength of photonic crystal-based nanofluidic structure according to Eq. (2). In order to deposit the Si_3_N_4_ layer matches with the designed Si_3_N_4_ thickness at 450 nm, the deposition rate of Si_3_N_4_ was carefully calibrated. The Si_3_N_4_ deposition process was processed in STS PECVD. The recipe for Si_3_N_4_ deposition was RF frequency 13.65 MHz, power 20 W, N_2_ flow rate (1960 sccm) 10%, SiH_4_ flow rate (80 sccm) 20%, NH_3_ flow rate (55 sccm) 10%, and chamber pressure 900 mTorr. In order to calibrate the deposition rate of Si_3_N_4_, a sequence of testing experiments had been done, as shown in Fig. 5b. The result from Fig. 5b shows that the deposition rate is almost constant, about 143 Å/min.

### CMOS-Compatible Fabrication Process

**Fig. 6:**
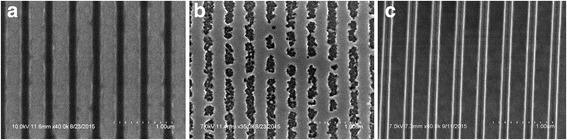
SEM pictures of grating patterns. **a** E-beam-patterned grating of PMMA on top of Cr layer, **b** Wet etching Cr with Chrome etchant. **c** Dry etching Cr with STS thermal ICP RIE

As Si_3_N_4_ is dielectric and transparent upon visible wavelength region, it is difficult to find the nano patterns with microscope. Chrome had been selected as hard mask for Si_3_N_4_ layer, which is much easier to pattern with Cr under e-beam equipment. The coating of 30 nm chrome was done by CHA SEC-600 e-beam Evaporator, with a coating rate of 1 Å/s under vacuum condition. Then, e-beam lithography was used for patterning nano structures on the sample wafer with PMMA. Firstly, the sample wafer was cleaned by acetone, IPA, DI water, and IPA in consequence, and then dried with N_2_ gas. Then, PMMA was spun coated on the wafer as photoresist with Headway Spinner under 3000 rpm, and then preexposed on a 110 °C hot plate for 1 min. Then, the PMMA-coated wafer was patterned by the Zenith e-beam lithography equipment, and the designed grating patterns were written on the PMMA layer. Then, the patterned PMMA wafer sample was post exposure at 220 °C for 1 min and cleaned, as shown in Fig. 6a. With the PMMA patterns as mask for Cr etching, there are two etching methods: dry etching and wet etching. Dry etching was using reactive ions such as chloride ions to react with Cr as selectively etching, and also the ions physical bombardment was used to etch the surface of PMMA and Cr simultaneously. Wet etching was using Cr etchant to etch chrome, which is much faster but it would destroy the sidewall of nano patterns. As Cr was used as hard mask, dry etching method was much more stable and conformal than wet etching method, and the Cr was etched on a Plasma Thermal SLR 770 ICP RIE. As shown in Fig. 6b, c, dry etching is anisotropic, so the shape of grating is uniform, and the fidelity of grating structure is quite well, while wet etching is isotopically, and it can cause damage to the grating patterns.

The ion gas used for Cr etching was Cl_2_, Ar, and O_2_. The chemical reaction process for dry etching was as follows:

$$ \begin{array}{l}\mathrm{Cr} + 2{\mathrm{O}}^{*} + 2{\mathrm{Cl}}^{*}\to\ {\mathrm{CrO}}_2{\mathrm{Cl}}_2\\ {}{\mathrm{CrO}}_x + \left(2- x\right)\ {\mathrm{O}}^{*}\to\ {\mathrm{CrO}}_2{\mathrm{Cl}}_2\end{array} $$

**Fig. 7:**
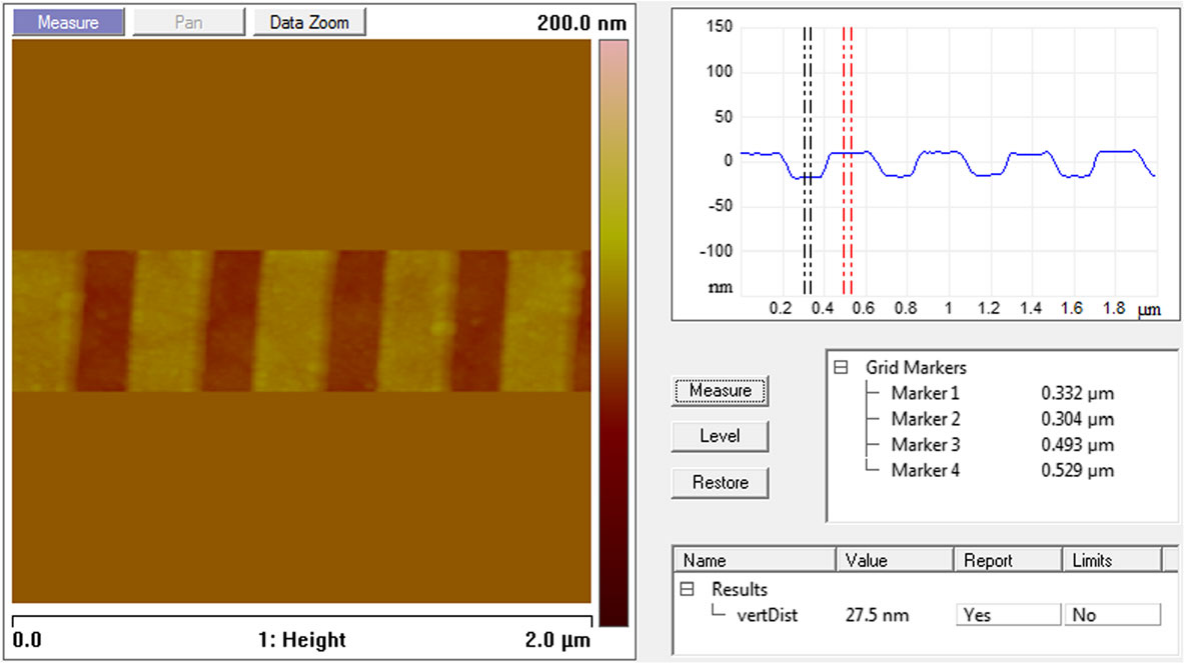
AFM picture of etched Cr layer profile

Since argon is an inertial gas, the accelerated argon gas has high momentum which can be used to bombardment of the Cr surface as physical sputtering. The recipe for chrome etching was ICP 100 W, RIE 250 W, pressure 50 mTorr, Ar 4 sccm, and O_2_ 6 sccm. The etch rate was about 4 nm/min. After Cr etching, the Cr pattern was used as hard mask for Si_3_N_4_ etching. As the etching rate between small features and buck samples was quite different, four 2 mm × 2 mm e-beam patterned samples were used for testing and calibrating. The dry etching result of Cr layer was shown in Fig. 7. The depth of the Cr layer is about 27.5 nm with grating pitch at 400 nm, and the profile of grating structures is quite uniform.

**Fig. 8:**
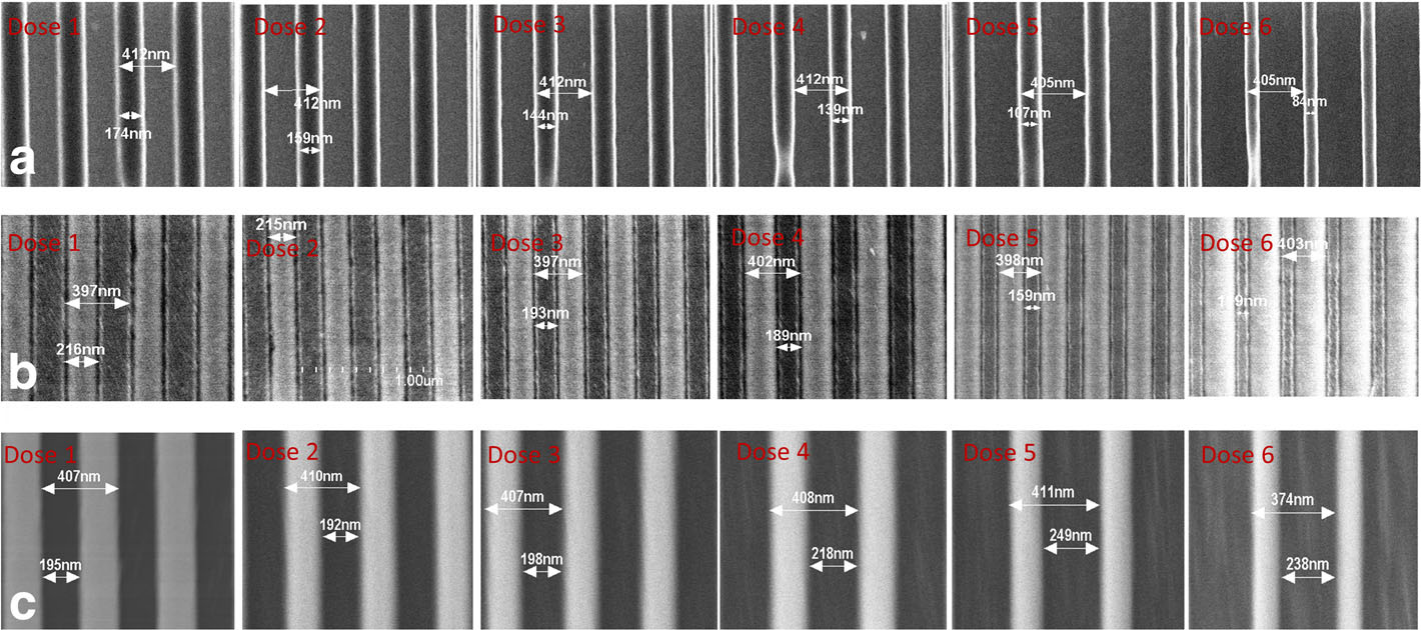
SEM pictures of dose-related PC grating structures, six different e-beam lithography doses were used for fabrication. **a** PMMA gratings patterned by e-beam lithography. **b** Cr gratings with patterned PMMA as photoresist. **c** Si_3_N_4_ gratings as guided mode layer for PC, and etched with Cr as hard mask

The equipment used for Si_3_N_4_ etching was STS Thermal Plasma ICP RIE. Since metal mask would contaminate the process chamber and it was hard to clean, this equipment was chosen specially for metal mask etching. The recipe for Si_3_N_4_ etching was Freon 14(CF4) 30 sccm, pressure 35 mTorr, RIE 95 W, ICP 300 W. The calibrated Si_3_N_4_ etching time was 4 min for 350 nm. In order to transfer the required feature to the Si_3_N_4_ layer, there were two different material layers that were acting as mask, PMMA layer, and Cr layer. As the grating feature was written on the surface of PMMA by e-beam lithography, the PMMA was used as mask for Cr etching. While the grating patterns transfer to the Cr layer, the etched Cr was used as mask for Si_3_N_4_ etching. Moreover, with the existence of undercutting effect, it was difficult to ensure the fill factor locates at 0.5. In order to obtain the desired fill factor and structure, a sequence of six different doses was used for e-beam lithography while writing on the surface of PMMA, as shown in Fig. 8a. Then, the dose-related samples were used for Cr etching and obtained SEM grating profiles, as shown in Fig. 8b. After the related etching of Cr and Si_3_N_4_ layer, six different Si_3_N_4_ grating patterns were sculptured, as shown in Fig. 8c.

**Fig. 9:**
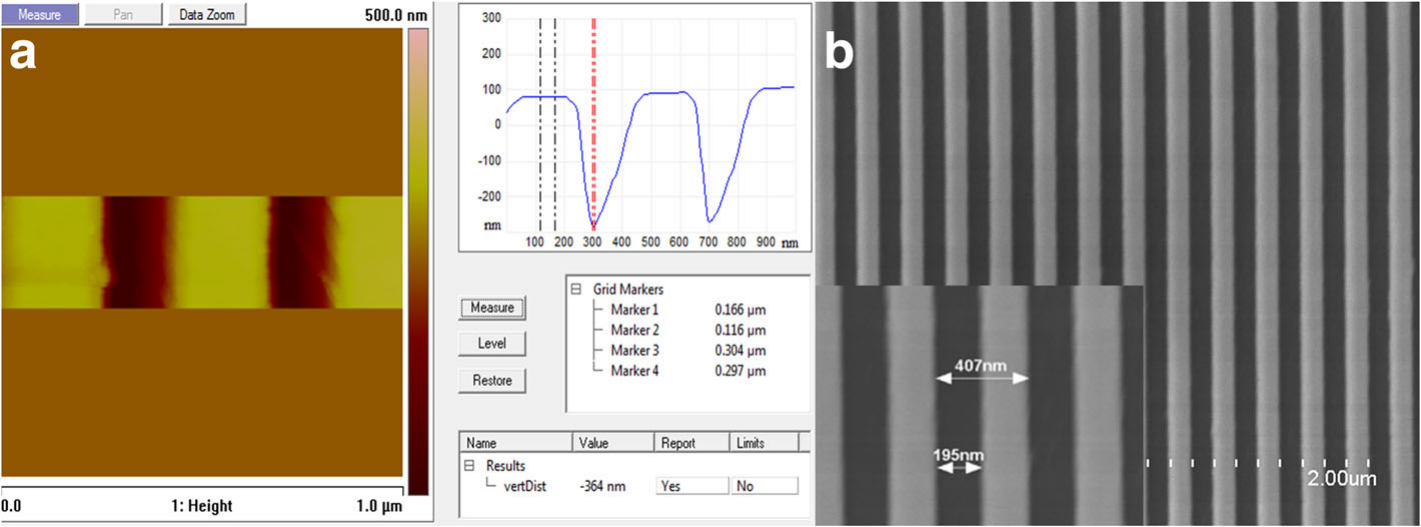
Inspection profile of Si_3_N_4_ gratings. **a** AFM image of Si_3_N_4_ grating depth and profile. **b** SEM image of Si_3_N_4_ gratings with periodic at 400 nm and fill factor 0.5

According to the fill factor calculation results, dose 1 had been selected for e-beam lithography and final experiment, and the result is shown in Fig. 9a, b. The inspected result from AFM is shown in Fig. 9a. From the AFM profile, it can be known that the grating pitch is located at 400 nm and the grating depth is about 367 nm. As the grating pitch was 400 nm with a fill factor 0.5, it was difficult for the AFM tip to touch the bottom of the grating and have a rectangular shape, so the profile looks like V-shape. The grating shape was detected under SEM, as shown in Fig. 8b which indicates that the sidewall of the grating is good enough.

After the etching of Si_3_N_4_, the PMMA and Cr remained on the sample need to be removed. The removing process was as follows: the sample was immersed in the acetone, and ultrasonic was used to speed the dissolve, and then the sample was rinsed by acetone, IPA, DI water, and IPA in sequence. Chromium etchant from Sigma-Aldrich was used to remove remained Cr. The wet etching speed was about 40 Å per minute at room temperature. With the fabricated grating structures, the inlets and outlets of PC-based nanofluidic structure were sculptured with photolithography, backside alignment, and etching in sequence. A 5-in. ferric oxide photo mask was fabricated and been used as photomask for photolithography. The photolithography process was as follows: (1) patterned Si wafer were cleaned by piranha process and O_2_ plasma for 1 min at 300 W: (2) prebake at 110 °C for 2 min; (3) cooling 30 s on the cooling plate; (4) descum with O_2_ plasma at 250 W, 150 mTorr for 1 min, (5) spin coating HMDS and SPR-220 in sequence at 3000 rpm for 30 s respectively, and the thickness of the SPR-220 will be 5 um; (6) soft bake at 60 °C for 2 min and 110 °C for 1 min; (7) backside alignment and exposure with i-line photolithography from EVG 620 aligner. The photolithography dose was 180 mJ/cm^2^ with vacuum contact and 50 μm separation distance, (8) 1:5 mixture of AZ 400 K and DI water as developer for exposed SPR-220; (9) the wafer was cleaned by DI water and N_2_ gas. With the SPR-220 patterns on the back of the patterned wafer, the inlets and outlets were fabricated by etch through the Si and SiO_2_ from the SPR-220 patterned side of Si wafer.

**Fig. 10:**
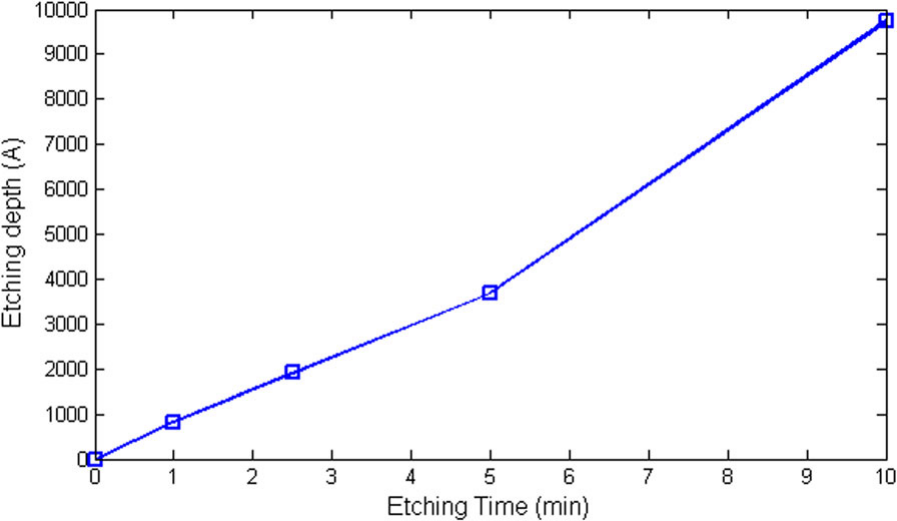
Etching rate of SiO_2_ with RIE etching method

The equipment used for Si etching was STS Pegasus ICP-DRIE, which has an etch rate of 20 μm/min with Bosch process. The whole Si layer etching process took 25 min, and the etching process would stop when the etching gases met the SiO_2_ layer. The etched Si pattern was used for SiO_2_ etching. Plasma Therm SLR-770 ICP RIE was used for the etching process, and the calibrated SiO_2_ etch rate was shown as in Fig. 10. The recipe used for SiO_2_ etching was CF_4_ 30 sccm, Ar 4 sccm, O_2_ 5 sccm, RIE 250 W, ICP 50 W, and pressure 30 mTorr. The RIE power was mainly used to break the chemical bond, while ICP power was aimed to enhance the plasma density.

**Fig. 11:**
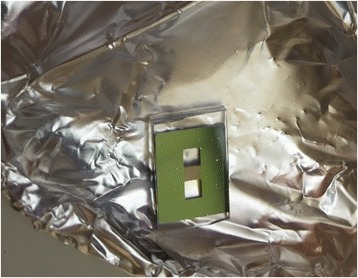
Prototype of PC-based nanofluidic structure

In order to fabricate the nanofluidic channel, a cladding layer was used to bond with the PC grating structure. Buck polydimethylsiloxane (PMDS) was made by mixing liquid PDMS and cross-linking agent. PDMS is a typical bonding method for polymer bonding, especially for rapid prototyping and used as plastic substrate. The buck PDMS and grating structure were treated with O_2_ plasma (300 W, 30 s) to make the surface hydrophilic. After the surface treatment, they were directly bonded with each other as shown in Fig. 11.

### Discussion

As PC’s grating period Λ, grating depth d_g_, and fill factor are important parameters to the final performance of PC-based nanofluidic sensor, maintaining the fabricated results conform to the designed structure parameters is of key importance. In this work, in order to keep the fabricated PC structure conform with the designed parameters, Si_3_N_4_ and SiO_2_ deposition rates had been calibrated and deposition thicknesses were carefully controlled. The obtained Si_3_N_4_ grating thickness is 364 nm which is very close to the designed d_g_ 350 nm, as shown in Fig. 9. Also, the measured PC grating period Λ 400 nm, which is equal to the designed grating period, as indicated from Fig. 7. To fabricate a uniform grating structure with fill factor 0.5, six different dose of e-beam process were used, and a uniform PC grating structure was yield according to the dose 2 process. A direct bonding method was used to realize nanofluidic channels between PC grating grooves and PDMS cladding layer. The bonding process is low cost and easy to implement, which is much convenient than traditional anodic bonding process.

This kind of structure is an improvement while contrast with PC-based microfluidic sensors, basically there are two kinds of applications: (1) Concentration detection: the reflected resonance wavelength of PC-based nanofluidic sensor will shift as the concentration of the liquid sample varies. There are a lot of applications, such as alcohol concentration detection, special protein concentration detection, small molecules, and even single molecule. It can be used for medical and industry applications. (2) Fluorescence enhancement: the enhanced electric field will improve the fluorescence efficiency when the incident light matches with the exciting wavelength range of the fluorescence dye. Since plenty of biological and medical applications are fluorescence based, the fluorescence enhancement can be used to improve the limit of detection for fluorescence-based experiments. The advantages of PC-based nanofluidic sensor have two basic aspects: (1) the volume of required sample is reduced to nanoscale, which can improve the efficiency of sample dose. (2) The nanofluidic channel can guarantee the detection area be confined around the evanescent region of PC structure, which can improve the sensor’s sensitivity. The function range of evanescent field is less than 400 nm, and it is the main region of enhanced electric field, which will interact with the fluid sample and obtain a resonance wavelength shift. With these unique performances, the PC-based nanofluidic structure sensor can be used for biomedical, chemical, and even clinic application applications in the near future.

## Conclusions

CMOS-compatible process of PC-based nanofluidic structure had been proposed, designed, and fabricated. The nanochannels have been created by polymer bonding between periodic gratings in PC and PDMS substrate. A detailed fabrication flow process was designed to guide the top-down fabrication process, and CMOS-compatible techniques had been used to realize the structure. The PC-based nanofluidic structure, with Si_3_N_4_ as its guided mode layer and SiO_2_ as its substrate, has a grating period Λ 400 nm, Si_3_N_4_ thickness d_0_ 450 nm, and grating depth d_g_ 350 nm. The resonance wavelength at 598 nm for higher order TE mode was selected for potential label-free detection. It is the first time that a PC-based nanofluidic structure has been proposed and realized with CMOS-compatible process. The enhanced electric field is located in the center area of nanochannels, which can be further benefit for analytes interaction experiments.

## Abbreviations

CMOS: Complementary metal oxide semiconductors
DI water: Deionized water
FDTD: Finite difference time domain
IPA: Isopropyl alcohol
PC: Photonic crystal
PDMS: Polydimethylsiloxane
PWV: Peak wavelength value
